# Survey of Schistosomiasis in Saint Lucia: Evidence for Interruption of Transmission

**DOI:** 10.4269/ajtmh.19-0904

**Published:** 2020-02-10

**Authors:** Janice Gaspard, Madelaine M. Usey, Merlene Fredericks-James, Maria J. Sanchez-Martin, Lydia Atkins, Carl H. Campbell, Paul L. A. M. Corstjens, Govert J. van Dam, Daniel G. Colley, W. Evan Secor

**Affiliations:** 1 Ministry of Health and Wellness, Castries, Saint Lucia;; 2 Integrated Life Sciences Program, University of Georgia, Athens, Georgia;; 3 Neglected Infectious Diseases, Pan American Health Organization, Washington, District of Columbia;; 4 Schistosomiasis Consortium for Operational Research and Evaluation, Center for Tropical and Emerging Global Diseases, University of Georgia, Athens, Georgia;; 5 Department of Cell and Chemical Biology, Leiden University Medical Center, Leiden, Netherlands;; 6 Department of Parasitology, Leiden University Medical Center, Leiden, Netherlands;; 7 Department of Microbiology, University of Georgia, Athens, Georgia;; 8 Division of Parasitic Diseases and Malaria, Centers for Disease Control and Prevention, Atlanta, Georgia

## Abstract

Saint Lucia at one time had levels of schistosomiasis prevalence and morbidity as high as many countries in Africa. However, as a result of control efforts and economic development, including more widespread access to sanitation and safe water, schistosomiasis on the island has practically disappeared. To evaluate the current status of schistosomiasis in Saint Lucia, we conducted a nationally representative school-based survey of 8–11-year-old children for prevalence of *Schistosoma mansoni* infections using circulating antigen and specific antibody detection methods. We also conducted a questionnaire about available water sources, sanitation, and contact with fresh water. The total population of 8–11-year-old children on Saint Lucia was 8,985; of these, 1,487 (16.5%) provided urine for antigen testing, 1,455 (16.2%) provided fingerstick blood for antibody testing, and 1,536 (17.1%) answered the questionnaire. Although a few children were initially low positives by antigen or antibody detection methods, none could be confirmed positive by follow-up testing. Most children reported access to clean water and sanitary facilities in or near their homes and 48% of the children reported contact with fresh water. Together, these data suggest that schistosomiasis transmission has been interrupted on Saint Lucia. Additional surveys of adults, snails, and a repeat survey among school-age children will be necessary to verify these findings. However, in the same way that research on Saint Lucia generated the data leading to use of mass drug administration for schistosomiasis control, the island may also provide the information needed for guidelines to verify interruption of schistosomiasis transmission.

Schistosomiasis, a parasitic disease caused by blood flukes, affects more than 200 million people globally. The infection is acquired through contact with fresh water that is inhabited by the parasite’s intermediate host, specific freshwater snails, and contaminated with human waste containing schistosome eggs.^1^ Consequently, this neglected disease primarily affects impoverished communities and is of major importance to global public health.^2^ Of the schistosome species that infect humans, *Schistosoma mansoni* is the only species transmitted in Caribbean nations and in Latin America.^3^ Infection with *S. mansoni* results in intestinal schistosomiasis, which can cause diarrhea, bloody stool, anemia, impaired cognitive development, and stunted growth when left untreated. The most severe cases involve periportal hypertension, ascites, and hepatosplenomegaly.^1,3,4^

In the mid-20th century, inhabitants of Saint Lucia, an island nation in the eastern Caribbean, demonstrated high levels of infection with *S. mansoni*. In the 1970s, some high-transmission areas demonstrated greater than 50% infection prevalence in all age groups, with children frequently exhibiting extremely high egg counts (> 1,000 eggs per gram feces) and hepatosplenomegaly.^5,6^ In addition, the snail intermediate host for *S. mansoni*, *Biomphalaria glabrata*, was widely distributed throughout water sources on the island.^5^

With the goal of reducing the rising infection levels, the Saint Lucian government partnered with the Rockefeller Foundation to enact a project to identify the optimal schistosomiasis control strategy for the island.^5^ When this partnership began in 1965, available schistosomiasis control methods included molluscicides, public health education, water and sanitation improvements, and selective chemotherapy. However, because combinations of these methods had been used, it was unclear which individual methods had contributed most to schistosomiasis control and provided the most cost-effective solution to reduce disease burden.^5^ The mountainous topography of Saint Lucia, which creates relatively secluded valleys with distinct watersheds between which travel was limited, offered a unique opportunity for the researchers to test the individual effectiveness of these different schistosomiasis control methods^5^ and subsequently to evaluate them in different combinations.

The cost-effectiveness findings from the Saint Lucia project were pivotal in establishing the current global strategy of preventive chemotherapy through mass drug administration with praziquantel for schistosomiasis control. Following the implementation of multiple control efforts, the prevalence of infection had declined to 9% by 1981.^6^ However, once the project with the Rockefeller Foundation concluded, few surveys to assess *S. mansoni* prevalence in Saint Lucia were conducted. In 2006, an epidemiological survey was conducted in 554 schoolchildren from the southern part of the island, and four cases (0.6%) of *S. mansoni* were found using the Kato-Katz stool microscopy assay.^7^ Between 2006 and 2015, primarily through mandatory stool screening of those in the hospitality industry, 36 individuals were diagnosed with schistosomiasis. Most of these were adults who were screened by thick stool smear. However, one 10-year-old boy was diagnosed with *S. mansoni* infection in 2010,^8^ suggesting that some level of transmission was still occurring later than 2000. Thus, although the infection rates appeared very low, the status of schistosomiasis transmission on Saint Lucia remains unclear.

In 2012, through World Health Assembly Resolution 65.21, the WHO called for nations endemic for schistosomiasis to adopt intensive control programs to achieve elimination of transmission where feasible.^9^ A limited number of studies in Morocco, Japan, Puerto Rico, and Egypt^10,11^ suggest that schistosomiasis transmission can be interrupted in endemic areas, but criteria to verify elimination have not been defined. The situation in Saint Lucia presents an opportunity to develop and evaluate possible approaches for verifying elimination. A first step is to assess the current state of *S. mansoni* infection on Saint Lucia.

## METHODS

### Survey population.

A school-based survey of children aged 8–11 years (grades 3 to 6) across all public primary schools was used to estimate schistosomiasis transmission. Primary education is mandatory and enrollment across Saint Lucia for the 2013/2014 school years approached 95%. Thus, children sampled from schools were expected to be representative of the primary school-age population of the country. Public schools were selected as they represented 94% of the total enrolled school-age population. The Ministry of Education reported a total enrollment of 16,628 children, with 8,985 students enrolled in grades 3 to 6, across 63 primary schools in the 2016/2017 school year. Primary schools are present across the entire island with some aggregation in urban areas. The survey was designed to detect a schistosomiasis prevalence of 1% with a 0.5% SD. The target sample size was adjusted to include a design effect of 1.5 and a 15% buffer to compensate for parents or children who declined study participation. Accordingly, it was estimated that 2,243 (1,950 total samples, +15% refusal) children should be included in the survey sample. This would represent approximately 25% of children enrolled in school between the ages of 8 and 11.

### Selection of children and sample collection in schools.

Before the survey, all parents and guardians were informed about its purpose, the sample collection process and testing, as well as the risks and benefits of participation via an official letter and a parent–teacher conference. If parents did not provide permission, their child was removed from the list of eligible children. Children were also informed of the aim and purpose of the survey and were provided with detailed information involving sample collection procedures. Children provided assent to participate and had a right to withdraw at any time. Participating students were given a flexi-pencil.

Sampling at individual schools was based on population proportional to size. A list with the names of all children in grades 3 to 6 was compiled. The first child was selected from the class register using a random number generator, then every fourth child (to represent 25% of total children) was selected until the required sample was drawn from each school. Children who participated were assigned a personal study identification code to protect their personal health information. A bathroom monitor labeled a plastic container with the child’s identification number, provided verbal instructions for fresh urine collection, and received the sample to ensure that the urine corresponded with the correct identification number. Finger stick blood samples were obtained by registered nurses using a safety lancet and collected on TropBio filter paper collection cards (CeLLabs, Sydney, Australia). Finally, a questionnaire about water usage habits and access to sanitation facilities was administered to each child by a student nurse. All samples and questionnaires were labeled with the child’s identification code and linked to the master sample list, by date and time of sample collection. Because it is not uncommon for children to attend school in an area different from where they live, their community of residence was noted to provide more accurate mapping of areas where transmission might have occurred.

### Circulating antigen testing.

Urine samples were transported to one of four nearby health posts in an insulated container. Commercially available point-of-care circulating cathodic antigen (POC-CCA) tests (Rapid Medical Diagnostics, Pretoria, South Africa batch #170316032 for the 2017 tests and batch #170622073 for the 2018 tests) were used to detect CCA from adult schistosomes according to manufacturer’s directions. Samples were scored as 0 (negative), trace, 1+, 2+, or 3+. Trace results were considered positive. Urine samples that yielded a positive result in the field along with a subset of urines that tested negative were stored at 4°C and subsequently sent to the University of Georgia (UGA) for retesting. Urine samples with equivocal or consistently positive results were sent to Leiden University Medical Center (LUMC) for up-converting particle-lateral flow circulating anodic antigen (UCP-LF CAA) assay as previously described.^12^

### Schistosome-specific antibody testing.

Filter papers containing the blood spots were dried, sorted, placed in plastic bags with desiccant, shipped to the CDC, and stored at −20°C until testing. For serologic testing, enzyme-linked immunosorbant assays were used to detect IgG antibodies specific for *S. mansoni*–soluble egg antigen (SEA).^13^ Immulon 2HB plates (ThermoScientific, Rochester, NY) were coated overnight at 4°C with 100 µL/well of 2 µg/mL SEA in 0.1 M sodium bicarbonate buffer (pH 9.6). Each dried blood spot (DBS) was eluted overnight in 250 µL of sample buffer (0.01 M PBS, pH 7.2, containing 0.3% Tween-20% and 5% nonfat dried milk) at 4°C on a shaker. Standards, controls, and samples were diluted to a 1:50 concentration in sample buffer and allowed to incubate on the plate at room temperature for 30 minutes. A standard curve of 0 to 500 arbitrary units (AUs) using pooled positive sera was included on each plate. All standards, controls, and samples were run in duplicate. Plates were washed five times using 0.01 M PBS, pH 7.2, containing 0.3% Tween-20. Mouse antihuman IgG conjugated to horseradish peroxidase (Southern Biotech, Birmingham, AL) at a dilution of 1:50,000 in sample buffer was added and allowed to incubate for 30 minutes. Following the incubation and plate washing, SureBlue™ TMB substrate (SeraCare Life Sciences, Gaithersburg, MD) that had been warmed to room temperature was added and incubated for 5 minutes before addition of 1 N sulfuric acid to stop any further reaction. The standard curve cutoff for this assay has been established at 40 AU but to be sure that all possible positives were identified, any samples with ≥ 30 AU were also tested by immunoblot against *S. mansoni* adult worm microsomal antigen for confirmation. Before Western blot testing, DBSs were eluted overnight in 500 µL of sample buffer at 4°C on a shaker. Immunoblots were conducted as previously described.^14^

### Ethical considerations.

The survey protocol was approved by the Saint Lucia Medical and Dental Council Ethics Committee, the Ministry of Education’s research approval committee, and the Pan American Health Organization’s Research ethics committee. The protocol was also reviewed by CDC and designated as a program activity. Local health and education authorities were also officially informed and agreed to participate. Any participant who tested positive would be given a referral to the nearest health center for treatment according to international standard treatment guidelines for schistosomiasis: 40 mg/kg praziquantel.

## RESULTS

### Survey population.

The survey took place in April and May of 2017. A total of 1,536 children were enrolled in the survey and provided responses to the questionnaire; 1,487 provided urine samples for testing, 1,455 provided fingerstick blood for immunologic analysis. The enrollment fell short of the targeted sample size of 2,243, but it still represents more than one of every six children in the eligible age range for every measure across the entire country. The rate of participation in the survey was similar for every school and area of the island.

### Circulating antigen testing.

Initial urine testing using the POC-CCA test was conducted in Saint Lucia by nurses and laboratory workers who had recently been trained on how to perform the POC-CCA. Of the 1,487 urine samples tested, 1,279 (86%) were scored negative, 59 (4%) were scored trace or positive, and 150 (10%) were scored “suspicious,” meaning that the reader was not comfortable scoring the test as a clear positive or a clear negative. Urine samples that yielded positive or suspicious results, along with some negative samples, were stored at 4°C until they could be shipped to UGA for confirmation testing.

A total of 307 urine specimens were selected, shipped, and received at UGA, but 116 of these specimens were deemed not suitable for additional evaluation because they were received without proper temperature conservation. Of the 191 specimens considered suitable for evaluation and tested at UGA, 94 (49.2%) were negative and 97 (50.8%) were positive by POC-CCA. Of the 97 positive samples, 76 (78%) were read as “trace.” If “trace” is considered a positive result, then 6.5% (97/1,487 urine specimens) would be the percentage of children on Saint Lucia with positive results.

After testing, the 191 urines by POC-CCA, UGA then shipped 111 of those specimens, including both trace-positive and negative specimens by POC-CCA, to LUMC. These specimens were then tested using the highly sensitive and specific UCP-LF CAA assay. Of the 111 specimens tested by the UCP-LF CAA assay, nine gave non-negative results: two were considered indecisive, six were very low positive, and one was a low positive.

### Schistosome-specific antibody testing.

A total of 1,455 DBS samples were collected for serological testing. Of these, 10 (0.69%) had ELISA results > 30 AU, suggesting potential exposure to the parasite at some point during their lives. A second ELISA test was performed on these 10 samples, as well as a confirmatory immunoblot (Table 1). None of the 10 samples retested by ELISA were immunoblot positive. In addition, none of the 10 children with ELISA results > 30 AU were positive by POC-CCA (Table 1). Seven of the urine samples from these 10 participants were clearly negative when tested on Saint Lucia and were, therefore, not forwarded to UGA for confirmation testing. The other samples (3/10) were scored “suspicious” during POC-CCA testing on Saint Lucia and subsequently tested negative by POC-CCA at UGA.

**Table 1:** Immunoblot and POC-CCA results of children with borderline or positive ELISA results

School	ELISA 1 reading	ELISA 2 reading	Immunoblot	POC-CCA in Saint Lucia	POC-CCA in the University of Georgia
School A: urban	166.3	343.9	negative	negative	–
School B: urban	37.1	30.1	negative	negative	–
School C: urban	31.8	27.8	negative	negative	–
School D: urban	117.9	187.7	negative	negative	–
School D: urban	38.4	46.8	negative	suspicious	negative
School D: urban	70.6	132.8	negative	negative	–
School E: rural	33.4	30.8	negative	negative	–
School F: rural	47.3	59.1	negative	suspicious	negative
School G: rural	36.3	25.3	negative	negative	–
School H: rural	39.4	47.3	negative	suspicious	negative

POC-CCA = point-of-care circulating cathodic antigen.

### Follow-up testing.

Of the nine children whose urine specimens tested indecisive, very low positive, or low positive by UCP-LF CAA at LUMC, eight had provided DBS. When these samples were tested for schistosome-specific antibodies by immunoblot, one had a faint positive result. This was the same participant who had tested low positive by UCP-LF CAA; however, this individual had a negative ELISA result (6.2 AU).

In May 2018, the Schistosomiasis Program on Saint Lucia collected follow-up urine and blood specimens from the nine children whose initial urine specimens had provided non-negative results by UCP-LF CAA. Because they were not confirmed schistosomiasis positive in 2017, they had not been treated with praziquantel. POC-CCA testing at UGA found that six of the nine specimens still tested as positive with trace readings. All nine repeat samples were negative by immunoblot and UCP-LF CAA testing.

### Questionnaire data.

Of the 1,536 children who answered the questionnaire, 1,384 (90.1%) reported that they had water piped into their homes (1,147, 74.6%) or housing plot (237, 17.1%; 151 had both, 86 had water piped to their plot but not into their homes). Of the 303 children who did not receive piped water at their households, 105 (34.6%) had access to water at a public standpipe, 46 (15.2%) reported using rainwater, and 41 (13.5%) reported using water from a spring. Ten children overall, including one child that did not have access to piped water at their house or plot, reported using surface water from rivers or ponds. With respect to contact with rivers or ponds, 40.9% (626/1,532) of the children reported swimming, 31.8% (419/1,316) reported wading, 18.9% (253/1,341) reported fishing, and 15.7% (239/1,526) reported collecting water from rivers or ponds when service from the water supply was interrupted. Overall, 48% (737/1,535) reported some contact with fresh water.

With respect to sanitation, 98.7% (1,516/1,536) of survey participants had access to some sort of improved toilet facilities. Access to a flush toilet in their home was reported by 1,264 (82.3%) of the children. An additional 238 (15.5%) reported using a toilet connected to a septic tank or latrine and 14 (9.1%) more said they had access to shared sanitary facilities.

## DISCUSSION

Considering the totality of the results from the series of tests performed in these surveys, we were unable to confirm infection with *S. mansoni* infection for any of the children who participated in the study. Although some children were positive by antigen testing, the proportion positive was consistent with the percentage of false positives expected when using the POC-CCA assay in areas with a very low prevalence of schistosomiasis, as is the case with Saint Lucia.^10,11^ The small number of children who had ELISA results near or above the cutoff were negative in the confirmatory immunoblot and had negative POC-CCA results. The one study participant who had a low positive result in the antigen tests and immunoblot was negative by ELISA and negative for both antigen and antibody tests on follow-up testing, suggesting that the initial results were false positives. Even if the child had been infected in 2017 and naturally cleared his infection by 2018, although the antigen tests may have turned negative, true antibody responses to worm antigens do not clear that quickly.^15^

The finding that none of the children were positive for *S. mansoni* infection even though almost half of them reported contact with fresh water suggests that schistosomiasis transmission may have been interrupted on Saint Lucia. However, additional work will be necessary to verify this finding. For example, a survey of adults on Saint Lucia, especially those with occupational contact with fresh water, would be important as persons with untreated infections can continue to excrete eggs for decades. Similarly, a systematic survey of snails in freshwater bodies is needed to determine whether appropriate intermediate snail hosts remain on the island, where they are located, and if any of them are infected. As part of the Rockefeller Project, competitor snails that are not suitable intermediate hosts for schistosomiasis transmission were introduced and successfully replaced *B. glabrata* in several locations.^16^ People living near bodies of water where *B. glabrata* remains would be more likely to have infection and could be more intensively surveyed as part of the verification process. Finally, a second serosurvey of school children is needed to confirm the 2017 results and strengthen the evidence against ongoing transmission.

Just as the earlier work on Saint Lucia was critical for elucidating the current global schistosomiasis control strategy using mass drug administration, these surveys have the potential to inform guidelines to confirm elimination of transmission that are currently under development. Along with showing negative results from human testing, the high level of access to clean water and sanitation could be important considerations for dossier preparation. Another important aspect of the work on Saint Lucia was the formation of a steering committee that included representatives from the health, education, and agriculture sectors under the coordination of the Ministry of Health and Wellness. Engagement by these stakeholders with a range of expertise and commitment to completing various assessments was necessary to carry out the 2017 survey and will be needed to successfully undertake the challenging process of demonstrating elimination. As with any disease elimination effort, proving a negative result is difficult. But, by building on the promising results obtained in this first countrywide survey of schoolchildren, it may soon be possible to verify the elimination of schistosomiasis transmission on Saint Lucia as well as provide a template for the process of schistosomiasis elimination in other countries.
